# Review: Organic nanoparticle based active targeting for photodynamic therapy treatment of breast cancer cells

**DOI:** 10.18632/oncotarget.27596

**Published:** 2020-06-02

**Authors:** Hanieh Montaseri, Cherie Ann Kruger, Heidi Abrahamse

**Affiliations:** ^1^Laser Research Centre, Faculty of Health Sciences, University of Johannesburg, Doornfontein 2028, South Africa

**Keywords:** photodynamic therapy, photosensitizers, nanoparticle delivery, targeting agents, breast cancer treatment

## Abstract

Targeted Photodynamic therapy (TPDT) is a non-invasive and site-specific treatment modality, which has been utilized to eradicate cancer tumour cells with photoactivated chemicals or photosensitizers (PSs), in the presence of laser light irradiation and molecular tissue oxygen. Breast cancer is the commonest cancer among women worldwide and is currently treated using conventional methods such as chemotherapy, radiotherapy and surgery. Despite the recent advancements made in PDT, poor water solubility and non-specificity of PSs, often affect the overall effectivity of this unconventional cancer treatment. With respect to conventional PS obstacles, great strides have been made towards the application of targeted nanoparticles in PDT to resolve these limitations. Therefore, this review provides an overview of scientific peer reviewed published studies in relation to functionalized organic nanoparticles (NPs) for effective TPDT treatment of breast cancer over the last 10 years (2009 to 2019). The main aim of this review is to highlight the importance of organic NP active based PDT targeted drug delivery systems, to improve the overall biodistribution of PSs in breast cancer tumour’s.

## INTRODUCTION

Breast cancer is a pervasive and common disease, which causes the second highest cancer-related death amongst women worldwide [1]. Mutations in genes such as; HER2, p53, CHEK2, BRCA1, and BRCA2 as well as environmental factors are the main contributing factors which cause breast cancer [2]. Current conventional therapy approaches to treat breast cancer include; surgery, radiotherapy or chemotherapy, which are used singularly or in combination. However, these conventional treatment forms often induce unwanted side effects in patients due to their no specificity and so damage healthy cells, as well as leave patients with long-term suffering and surgical scarring [3].

In spite of the fact that efforts have been intensified to mitigate the side effects caused by conventional breast cancer treatments, progress in controlling this complex disease has been difficult due to the inadequate understanding of its basic biochemistry, which is the main effector of its development [4]. Most current chemotherapeutic agents have a low molecular weight and high pharmacokinetic volume of distribution, so they are cytotoxic, however easily excreted [5]. Therefore, high chemotherapeutic drug concentrations are required to be administered to patients, in order to induce cytotoxicity in tumour cells, prior to excretion, however these high concentrations (and lack of drug targeting specificity) also induce significant toxicity in healthy cells and so unwanted side-effects in patients is inevitable. In addition, when conventional drug agents are administered alone, a lack of specificity to targeted cells also hampers their uptake and so hinders the overall effectiveness of the treatment [5].

In this sense, nanoscience’s which deal with the development of new nanoparticle (NP) active targeting drug delivery systems is an emerging field. These systems enable the specific delivery of a drug to targeted cancer tissue and so consequently improve drug uptake efficacy and overall treatment outcomes, as well as minimize unwanted side effects and sometimes even allow for controlled drug release rates [5]. Thus, novel unconventional NP drug system-based treatment approaches are urgently needed for the improved treatment (as well as detection) of primary and metastatic breast cancers, in order to limit unwanted side effects, as well as enhance the overall survival rate of patients. The ultimate strategy of NP active targeting drug delivery systems is to obliterate the tumour cells, while sparing the normal cells, with improved treatment outcomes.

Photodynamic therapy (PDT) is a promising non-invasive unconventional treatment for a variety of diseases, including breast cancer. It involves the administration of a passive tumour-localizing photosensitizer (PS), followed by localized tumour irradiation at a specific wavelength of light, which in turn excites the PS. The excited PS then transfers its energy to tumour localized molecular oxygen, which causes cytotoxic reactive oxygen species (ROS) and singlet oxygen (^1^O_2_) to be generated, which in turn oxidize surrounding cellular macromolecules and so destroy tumour cells [6].

Among the broad spectrum of light, near infrared (NIR) window (750–1700 nm) enjoy low absorption and low scattering and deep tissue penetration together with low auto-fluorescence from biological tissues which can be applied for biophotonic imaging [7, 8].

Thus, the integration of nanoscience’s and PDT opens a new window of interest in the exploration and functionalized NPs for the targeted drug delivery of PSs to breast cancer cells, with high selectivity and specificity, in order to diminish unwanted side-effects on healthy cells, however enhance and overall improve treatment outcomes, as to ensure patient survival.

Therefore, this review comprises of an overview of scientific peer reviewed published literature in relation to functionalized organic NPs, for the effective targeted PDT (TPDT) treatment of breast cancer, which have been studied over the last 10 years (2009 to 2019). The main focus of this review is to highlight the importance of NP active PDT targeting drug delivery systems to improve the overall biodistribution of PSs to desired breast cancer tumour locations. This review should open new research initiatives and avenues for improved PDT breast cancer treatments outcomes, since current research seem to be solely focused on PS drug dosing optimization, instead of considering investigating specifically enhanced NP drug delivery systems for optimal treatment.

## CURRENT BREAST CANCER DIAGNOSIS AND TREATMENT OPTIONS

### Breast cancer diagnosis

Breast cancer is the second most common cancer in the world and currently affects 1 in 8 women [9]. The most common types of breast cancer are ductal carcinoma *in situ*, invasive ductal carcinoma, and invasive lobular carcinoma. Carcinomas are tumours which originate in epithelial cells that line tissues and organs throughout the body [10]. In order to thoroughly investigate any breast abnormality and diagnose breast cancer a “triple assessment” needs to be performed, which consists of a clinical examination, imaging, and cytology [11]. Each individual test complements the other and combined results allow for an accurate diagnosis [11].

### Conventional breast cancer treatment

Current conventional breast cancer treatment comprise of a variety of methods—surgery, radiotherapy, and chemotherapy—which may be applied to a patient singularly or in combination depending on the stage and type of breast cancer diagnosed [12].

Breast-conserving surgery (BCS) comprises of mastectomy and lumpectomy. Lumpectomy is considered as an alternative to mastectomy. Mastectomy surgery removes the entire breast, whilst lumpectomy removes the cancerous cells and preserves the breast as much as possible [13]. Mastectomy takes longer and is more extensive than lumpectomy, while lumpectomy is less invasive. Breast cancer represents an overall higher risk of recurrence [14]. It has been reported that 60–75% of breast cancer patients receive partial mastectomy as initial treatment [15]. Despite the fact that post mastectomy radiotherapy (PMRT) decreases local recurrence in patients with locally advanced breast cancer [16], it produces a poor cosmetic outcome in women with breast reconstruction [17]. In addition, although lumpectomy followed by radiotherapy is an effective approach for the treatment of early breast cancer, up to 60% of patients need additional surgery due to positive margins [18, 19]. Additionally, repeating of BCS alone can cause markedly high local failure rates, ranging between 7–50% [20].

Even though radiotherapy can induce significant reduction in breast cancer recurrence and mortality after BCS or a mastectomy [21], the overall benefit of radiotherapy is reduced by high mortality risk of heart disease in patients [22]. Although chemotherapy drugs can diminish mortality and recurrence of breast cancer, they are cardiotoxic and they increase the risk of heart failure and cardiomyopathy in patients, as well as induce debilitating side effects in patients [23, 24]

Therefore, the challenge is still to find minimally invasive techniques for early diagnosis and treatment of breast cancer.

### Unconventional breast cancer treatment: photodynamic therapy

Photodynamic therapy (PDT) is an unconventional substitute for breast cancer treatment, where cancer cells are destroyed, when a non-toxic PS is passively absorbed by tumour cells and becomes activated at a specific wavelength of light to produce ROS and ^1^O_2_, which in turn destroy cancer cells [6].

**Figure 1:**
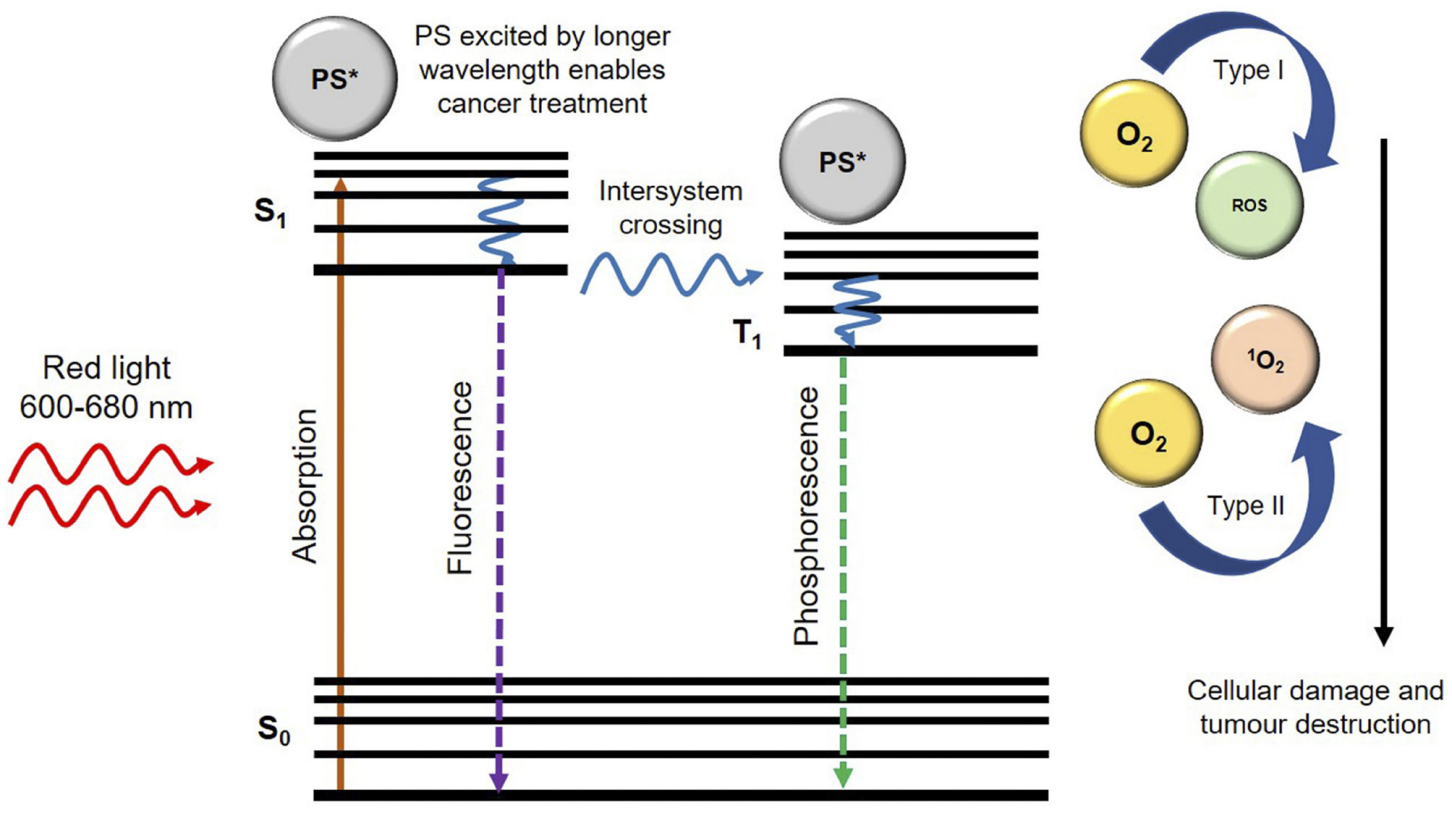
Generation of reactive oxygen species (ROS) and singlet oxygen (^1^O_2_) upon irradiation of photosensitizer (PS) with an appropriate wavelength of light.

Thus, PDT consists of three phases: excitation of PS molecules; at a specific wavelength of light, generation of cytotoxic oxygen, and final tumour cell death [25]. The chosen wavelength of the light (generally within the near infra-red range) usually coincides with the maximum absorption wavelength of the PS drug [26]. These PS drug molecules then react with free available molecular oxygen (O_2_) within cancerous cells, and so produces cancer-killing oxidizing molecules (ROS and ^1^O_2_) which cause irreversible damage to target cancer cells (Figure 1) [27].

In general, PDT can be considered a far safer breast cancer treatment for patients than conventional treatments, such as chemotherapy or radiotherapy, since the cancer-killing oxidizing molecules it produces are strictly localized in tumour cells only and so normal healthy tissues and cells remain unharmed [6]. Thus, the overall main advantages of PDT over conventional treatment methods comprise of a far more localized form of treatment, is less invasive, can be repeated without cumulative toxicity, fewer secondary effects, with excellent functional and cosmetic results, as well as improved quality of life of the patients [6, 28]. With reference to Supplementary Table 1, a summation of various PS chemical structures and absorption/activation wavelengths of light for PDT treatments of breast cancer can be observed.

Porphyrin-, cyanine-, pthalocyanine-, and chlorine-type PSs are generally applied to breast cancer due to their strong absorptions and effective PDT therapeutic window index of activation in the red or near-IR wavelengths [29]. Additionally, these PS types have well-defined compositions, with stable formulations, that can be administered intravenously [29]. Currently, chlorines (tin ethyl etiopurpurin [30], mono-L-aspartyl chlorin [31], and verteporfin [32]) and texaphyrin (motexafin lutetium [30]), which are expanded porphyrins with a penta-aza core are being investigated in clinical trials for breast cancer treatment [29, 33, 34].

Hitherto, paclitaxel albumin-stabilized nanoparticle formulation (nab-paclitaxel) known as Abraxane^®^ has been approved for the treatment of metastatic breast cancer which acts as an inhibitor of cell division [35]. The inhibition effect of paclitaxel on healthy cells, its poor solubility and toxicity due to cremophor EL (polyethoxylated castor oil) may restrain the application of this drug for treatment of patients [36]. One research study demonstrated that nab-paclitaxel in combination with S-1 (tegafur + 5-chloro-2.4-dihydrooxypyridine + oteracil potassium) is a promising therapy for the treatment of patients with HER-2 negative breast cancer [37].

Although doxorubicin plays a pivotal role in the treatment of adjuvant and metastatic breast cancer, bone marrow depression and cardiotoxicity have been considered as the major side effects of this formulation [38]. It was reported that liposomal doxorubicin and pegylated liposomal doxorubicin enjoy similar efficiency without the toxicities of conventional doxorubicin [38–40].

However, it is crucial to emphasize that the central challenge of PDT is how to deliver PSs efficiently to the cancerous cells in order to improve cellular uptake and overall treatment outcomes. Since, the efficacy of ROS generation for tumour damage is highly dependent on the uptake of the PS in tumour cells, as well as the PS ability to overcome a patient’s biological barrier [41]. Furthermore, some of currently used PSs including porphyrin derivatives, napthalocyanine, phthalocyanines and chlorines have non-specific distribution and slow excretion from the body, which lead to the detrimental side effects [42]. In addition, in view of the hydrophobic properties of PSs, they require dispersion in an emulsion or the use of a delivery system to carry the drugs to the biological media [43]. In this context, the utilization of nanocarriers to administer the drugs to the desired target can circumvent this issue [26].

Therefore, targeted PS uptake and NP delivery in tumour cells is a crucial factor in PS uptake studies, since non-nano targeted drug delivery mechanisms, allow for minor amounts of PSs to passively accumulate in tumour cells (due to the enhanced permeability and retention (EPR) effect) and the remainder is either destroyed by immune barriers or distributes into healthy tissues, causing unwanted side effects and poor treatment outcomes [41]. Thus, the ability of PSs to be selectively switched on in the tumour microenvironment or be able to respond to biological stimulus inside cancer cells is of great importance in PDT PS uptake assays [44]. Therefore, to improve the efficacy of PDT cancer treatment, research is currently focused on the development of specific receptor-based PS-nanocarrier platform drugs, which promote the active uptake and absorption of PSs in tumour sites only, as well as are able to avoid immunological barriers, therefore preventing unwanted side effects and overall treatment enhancement.

## PDT MECHANISM OF CANCER CELL DEATH

PDT mechanism of cell death is a two-stage process whereby a PS is administered to a patient and it is then activated by a light at a specific wavelength. The physiochemical properties and charge of a PS play a significant role in this process. Hydrophilic PSs interact with albumin and globulins, whilst hydrophobic PSs tend to bind to low-density lipoprotein (LDL) receptors [45]. Whereas, anionic PSs tend to collect in cellular lysosome organelles, cationic molecules are absorbed by the mitochondria [45].

**Figure 2:**
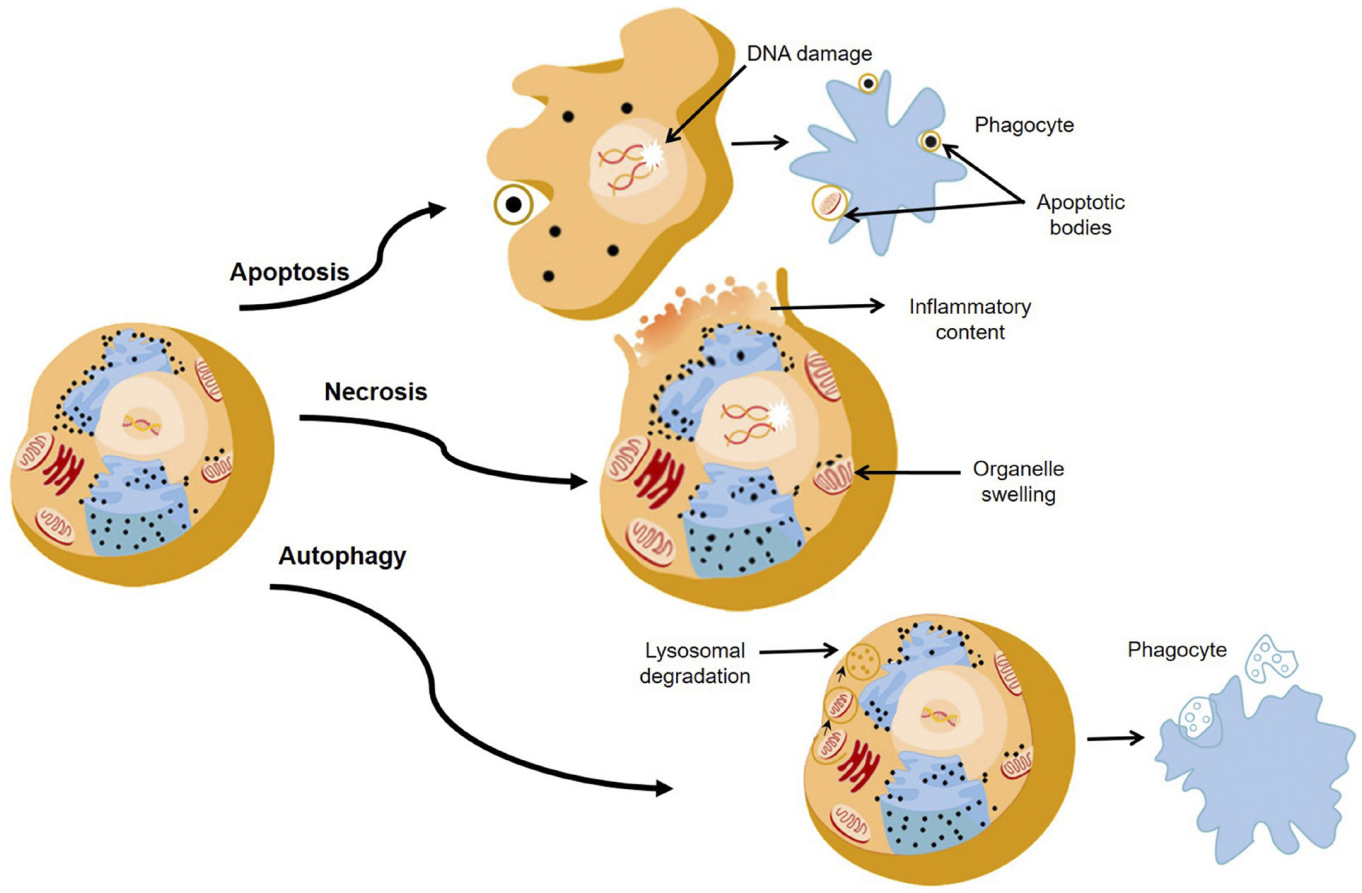
The major pathways of death cells through apoptosis, necrosis, and autophagy.

When a PS is activated at a specific wavelength of light, three main cell death pathways, namely apoptosis, necrosis, and autophagy are generally induced (Figure 2) [46–48]. However, the extent of PDT induced cell death is affected by different factors, such as the type of PS, its subcellular localization and uptake concentration, its physical-chemical features, the concentration of cellular oxygen, as well as the wavelength and intensity of the light that is applied [49]. Additionally, it must be noted that in some cases tumour cells can become sensitized to PDT and sometimes PDT can interfere with the cytoprotective molecular responses in cells, rendering it in effective [46].

In PDT, PSs act as catalysts which absorb visible light and with cellular oxygen produce highly ROS and ^1^O_2_. The basic mechanism of PDT relies on the absorption of light by a PS which in turn becomes excited. An excited electron from the PS then moves to a short-lived excited state, followed by intersystem crossing. Then the excited electron changes its spin to produce a longer-lived triplet state. The triplet state PS then transfers its energy to ground-state triplet surrounding oxygen, which in turn produces a range of ROS such as hydroperoxides, superoxide, or hydroxyl radicals, for Type 1 pathways and singlet oxygen (^1^O_2_) for Type II pathways [50] (Figure 1). These ROS can kill cancerous cells via apoptosis, necrosis, and autophagy pathways (Figure 2) [50, 51]. Both PDT type pathway reactions may occur singularly or simultaneously, however most PSs generally favour Type I pathways of ROS generation, followed by apoptotic cell death in cancer cells [41].

## NANOTECHNOLOGY FOR BREAST CANCER

Intensive screening and advanced treatment regimens have reduced the occurrence of breast cancer, with improved longevity of patients. However, many challenges remain in cancer theranostics such as minimizing the adverse effects, reducing off-target toxicity and the lack of a therapeutic target in relation to cancer drugs administration and adsorption [52]. The utilization of nanotechnology has emerged to overcome these obstacles. Nanotechnology involves the application of small particles, which have diameters in the range of 1–100 nm, allowing them to interact with cancer tumours at an intracellular level [53].

The combination of nanotechnology and cancer drugs with cellular and molecular techniques, has allowed for the development of satisfactory cancer diagnostic and therapeutic designs. NPs smaller than 50 nm can easily enter most cells and those smaller than 20 nm can circulate through blood vessels [54]. Thus, NPs can be used as drug delivery vehicles of chemotherapeutic agents into cancerous cells, while leaving healthy cells unaffected [54].

NPs have been investigated in order to enhance PS drug delivery in PDT, since they promote passive tumour PS drug uptake due to the EPR effect [55]. Furthermore, due to the small size of NPs they are easily absorbed by tumour microvasculature, allowing for enhanced PS drug accumulation [55]. Additionally, NPs exhibit a large surface area to volume ratio, meaning they can support a large PS drug load quantity, allowing for a higher concentration of the PS to be absorbed by tumours [55]. Moreover, NPs can mimic biological molecules and so often go by undetected by the body’s biological barriers, thus they protect PS drugs from immunological destruction [55]. Lastly, PDT studies have noted that the conjugation of PS drugs to NPs overall improve the stability, solubility, permeability and absorption of PSs and so the overall cancer PDT treatment regime is enhanced [55].

However, current research has moved onto investigating NPs which have been functionalized with various active targeting moieties, in order to allow for active targeting to occur and so allow for even higher PS drug loads to selectively accumulate in target cancer cells, with far more improved PDT treatment outcomes [41].

Overall, a great number of properties allow for organic NPs to be well suited for cancer diagnosis and therapy that distinguish them from nucleic acid therapeutics as well as inorganic NPs and their molecular conjugates. These unique properties include small size, duration of effect, payload density and surface allowable functionalization properties for effective PS drug targeting [56], which all have been summarized in Table 1.

**Table 1:** Important properties of organic nanoparticles

Organic NPs	Properties	Ref.
Polymeric NPs/micelles	Biocompatible, non-toxic, biodegradable, enhanced pharmacodynamic and pharmacokinetic properties	[57, 58]
Block copolymer micelles	High loading capacity, carrier of water insoluble drugs, protection against degradation, drug stability improvement,	[57, 59]
Liposomes	Carrier of neutral, hydrophilic and hydrophobic drugs, improved biocompatibility with PEG, suitable for passive and active targeting	[3]
Virus-like and albumin NPs	Water solubility, low immunogenicity, high tumour penetration and distribution, low cost production, high stability	[59, 60]

## ORGANIC NANO TARGETED PHOTODYNAMIC THERAPY FOR BREAST CANCER

The engagement of organic NPs in PDT has been established as an important overall therapeutic enhancement strategy, in order to induce improved cancer tumour cell death, since NPs act as selective PS drug transporters and energy converters within the PDT process [61]. Various active and passive organic nano targeted drug delivery approaches have been developed to effectively enhance PS drug uptakes in tumour cells. Although both processes rely on the circulation and localization of the PS to enhance PDT, active targeting moieties incorporated with NPs can enhance the accumulation of PSs very selectively into specific target tumour cancer cells [41].

### Nano passive PDT targeting strategy

The passive PDT targeting strategy enables the natural distribution of PSs into tumour cells via the EPR effect, as discussed previously. The main drawbacks of passive targeting pertains to the fact that small amounts of the PSs more selectively internalize and accumulate into cancer tissue, however some of the PSs can distribute in normal tumour surrounding cells causing unwanted side effects [41, 61]. In this regard, an active nano PDT targeting strategy, is currently being investigated in PDT in order to raise tumour PS uptake and improve specific sub-cellular localization.

### Nano active PDT targeting strategy

**Figure 3:**
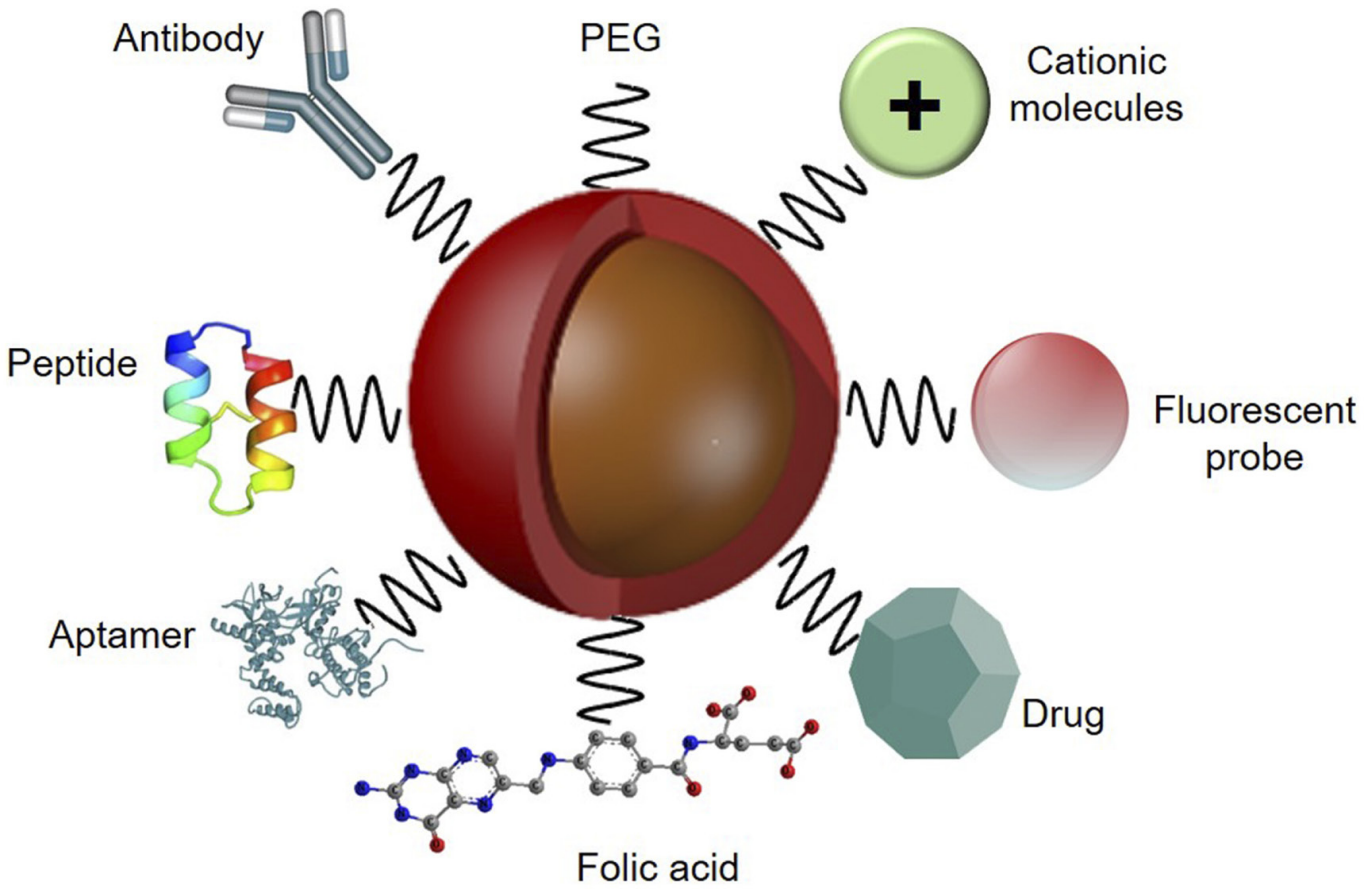
Functionalized nanoparticle platform with various targeting ligand agents for active PS delivery in PDT applications, with PEG to improve biocompatibility and an imaging fluorescent probe to monitor PS specific tumour cellular uptake.

The active organic nano PDT drug delivery strategy has emerged to preclude the limitations of passive targeting strategy, using specific cancer tumour targeting agents. Monoclonal antibodies (mAb), aptamers and nucleic acid, as well as small molecules, peptides, or their fragments, and nucleic acids have been emerged as tumour targeting ligand agents to functionalize the surface of nanoplatforms [61] (Figure 3). These homing ligands are attached to the surface of NPs, which then enable the active binding of the nanocarrier PS conjugate to overexpressed cancer cell receptors, such as epidermal growth factor receptors (EGFR), folate receptors (FR), transferrin-receptors (TfR), CD44 or CD22, to enhance overall PS cellular uptake and localization [61].

The specific active target overexpressed receptors for breast cancer cells include human epidermal receptor (HER), folate receptor, transferrin receptor and glycoproteins [61]. The human epidermal receptor-2 (HER-2) is the most widely studied and utilized overexpressed targeting receptor for therapeutic applications in relation to breast cancer [62]. The homing ligand targeted to HER-2 overexpression in breast cancer, is the anti-HER-2 mAb, which is mostly conjugated onto NP platforms for the active targeting PS delivery in PDT breast cancer applications [63]. However, it must be stated that the prohibitive expense of synthesis, large size, sensitivity of mAb-based NPs to environmental encounters and immunogenic properties may restrain their applications in clinical trials [64]. Thus, researchers often consider investigating aptamers, which are a second class of target moieties comprising of single-stranded RNA or DNA sequences of oligonucleotides, since they exhibit decreased immunogenicity, small size, stability and good bio-distribution, making aptamer-based nanotherapeutics far more suitable for medical testing [65].

Additionally, the incorporation of specific ligands into nanoformulations to promote the release of PSs into cancer cells via receptor-mediated endocytosis, while constraining the non-specific transport to the normal cells is also a promising focal approach of current research [66]. Some currently used receptor-mediated endocytosis based homing ligands consist of either transferrin based nanoplatforms, folic acid which overexpresses folate receptors, tumor-homing peptide—CREKA (Cys-Arg-Glu-Lys-Ala)-based nanoformulations or peptides belonging to the family of natural antimicrobial peptides (AMPs) [61].

In this review, the latest developments in the utilization of multi-functionalized organic NPs for only the active PDT targeting strategy of breast cancer, which have been developed over the last 10 years are discussed.

## FUNCTIONALIZED ORGANIC NANOPARTICLE-BASED ACTIVE TARGETING PDT STRATEGIES FOR BREAST CANCER

In order to enhance water solubility and accumulation of PS drugs into target cancer cells, they can be encapsulated into organic polymeric or lipid-based nanocarriers. Polymeric NPs/micelles, block copolymer micelles and liposomes together with naturally occurring NPs or biological NPs are potential candidates for organic NP PS carriers in PDT.

### Polymeric nanoparticles/micelles

Polymer-based NPs are fabricated from biodegradable or non-biodegradable materials. They are sometimes encapsulated in an oily or aqueous core surrounded by a polymeric shell. The main merits of polymeric NPs is their small size, surface structure and release rate of a PS, which can be fine-tuned by using appropriate materials [67, 68].

PSs can be loaded into polymeric/micelle NPs via covalent binding, encapsulation or post loading [49]. PSs can also be encapsulated into the core or shell of these NPs through electrostatic or hydrophobic interactions and hydrogen bonding [49]. Hydrophobic PSs are generally entrapped into these NPs via a hydrophobic interaction between the PS and the polymer itself [3].

Methylene blue (MB)-conjugated polyacrylamide (PAA) NPs coated with F3 peptides were proposed for the *in vitro* PDT treatment of MCF-7, 9L, MDA-MB-435, and F98 [69]. The F3-targeted MB-conjugated PAA NPs demonstrated a 4 to 5 times stronger efficiency to 9L and MDA-MB-435 relative to MCF-7 cells. According to the MTT assay, no dark toxicity was observed for the four cell lines in the absence of illumination, however upon illumination the cell death induced by PDT enhanced with irradiation time and NP dose. The PDT experiments with illumination at 647 nm depicted remarkable less cell death for MCF-7 than when compared to the other cell lines owing to the low levels of nucleolin receptors on the surface of these cells, which resulted in the low uptake of the NPs [69].

Regarding drug resistant breast cancers, chemo-photodynamic combination therapy has the capability of enhancing treatment efficiency. In this regard, tLyp-1 peptide-functionalized TPGS-PLA NPs (tLyp-1-NP) were synthesized by incorporating chlorine e6 PS in the shell and the chemo-drug doxorubicin (DOX), in the core of D-α-tocopheryl polyethylene glycol 1000 succinate-poly(lactic acid) (TPGS-PLA) NPs, with tLyp-1 targeting peptide on its surface [70]. The NPs indicated high *in vitro* cellular uptake and strong cytotoxicity for umbilical vein endothelial cells (HUVEC cells) and doxorubicin-resistant human breast adenocarcinoma cells (MCF-7/ADR cells) after irradiation at 660 nm. Compared with the NPs without DOX, the IC50 of NP and tLyp-1-NP in the absence of laser lessened by about 31.5 and 75.6 fold respectively, whilst after irradiation, the IC50 of tLyp-1-NP was 6.6 times lower than that without laser, because the overall ROS generation has enhanced cytotoxicity through endolysosome escape and PDT [71]. The *in vivo* targeting efficiency of tLyp-1-NP at 24 hrs post-administration exhibited superior accumulation and selectivity through the targeting ligand tLyp-1 peptide, as well as a time dependent accumulation of the targeted NPs with the maximum fluorescent intensity being observed within the breast cancer tumour. The mice tumors treated with the nanohybrid in the presence of laser showed slow growth from day 1 to day 4, and a gradual decrease in the later days [70].

Boron dipyrromethene (BODIPY) derivatives are suitable PSs for PDT which have gained much importance in recent years. Their high chemical stability and photostability, with high molar absorption coefficients, as well as easy core modification with tunable optical and photophysical properties are the main features of BODIPY [72]. In this context, mannose-functionalized PS NPs were utilized as selective internalized NPs for the treatment of MDA-MB-231 breast cancer cells [73]. The PS delivery system was based on BODIPY PS conjugated with adamantane (Ad) units (BTA). Heptamannosylated β-cyclodextrin (CD-Man_7_) was then immobilized onto the surface of BTA to stabilize the system in aqueous media (BTA@CD-Man_7_). The conducted experiments demonstrated that BTA@CD-Man_7_ NPs disassembled in breast cancer cells lysosome after internalization and no dark cytotoxicity was found for BTA@CD or BTA@CD-Man_7_. The cell viability efficiency of BTA@CD was reported to be 89%, while less than 9% viability was recorded for BTA@CD-Man_7_ after 30 min exposure to 665 nm light. *In vivo* treatments also confirmed the effectiveness of PDT with BTA@CD-Man7 as the breast cancer tumour growth was notably inhibited in a mouse model treated with BTA@CD-Man7 and irradiation when compared with BTA@CD and irradiation. Furthermore, the tumour weight in the mice treated with BTA@CD-Man7 plus irradiation was remarkably smaller than control groups [73].

In another study, a three-arm distyryl BODIPY derivative was conjugated to mannose targeting agent (BTM) and it was then co-assembled with Tween 80 to form nanomicelles (BTM-NMs) [74]. The as-prepared nanomicelles were applied for the PDT treatment of MDA-MB-231 breast cancer cells, with 665-nm LED light irradiation. In this study, glucose-functionalized nanomicelles (BTGNMs) were prepared as a control sample. It was observed that both MDA-MB-231 cells and MCF-10A cells had low dark cytotoxicity towards the nanomicelles. In addition, MDA-MB-231 cells treated with BTM-NMs showed significantly lower cell viability as contrast to the BTG-NMs [74].

The fabrication of nanostructured materials via host-guest interactions is a promising strategy to program hydrophobic PS drug into cancer cells. β-Cyclodextrin (β-CD) is a broadly used host molecule, which contains a hydrophobic interior for quest molecules and a hydrophilic exterior to intensify the drug solubilisation [75]. Programmed PS supramolecular nanomicelles, with a dual targeting ability were developed to scrutinize the PDT effect on an *in vitro* MCF-7 cell model [76]. Chlorin e6 (Ce6) was conjugated to the host molecule β-CD (CD-Ce6). The designed peptide adamantine-CGKRK-GFLG-EE-HAIYPRH(T7) and CD-Ce6 were then combined to prepare self-assembled nanomicelles of CD-Ce6/CGKRK-GFLG-T7, where Ce6 served as the hydrophobic core and the peptide performed the hydrophilic shell. The cellular uptake images confirmed the successful internalization of CD-Ce6/CGKRK-GFLG-T7 by MCF-7 cells. In fact, the presence of transferrin receptor (TfR) on the surface of the breast cancer cells facilitated the specific binding affinity of the nanomicelles through T7. It is also imperative to note that CD-Ce6/CGKRK-GFLG-T7 was programmed to accumulate within the mitochondria through enzyme-induced cleavage of the GFLG. After light irradiation, breast cancer cells treated with CD-Ce6/CGKRK-GFLG-T7 demonstrated 31.39% apoptotic cell death, while 18.48% apoptotic cell death was reported for the CD-Ce6/CGKRK-GGLG-T7 [76].

Glycopolymeric, glycodendrimers and glycoclusters materials have been introduced within breast cancer targeting PDT in order to enhance the binding affinity of the nano drug carrier to carbohydrate receptors. In one study conducted by Han *et al.* a simple procedure was employed to fabricate core–glycoshell theranostic nanomaterials [77]. Fluorescent glycoprobes were utilized as targeting and imaging agents, while poly(3-hexylthiophene-2,5-diyl) (P3HT) nanodots acted as a therapeutic material for PDT and as a vector. Poly(styrene-co-maleic anhydride) (PSMA) was also conjugated to the P3HT nanodots so as to enrich the water solubility. In addition, galactose (Gal)-dicyanomethylene-4H-pyran (DCM) dye and mannose (Man)-DCM were self-assembled with the P3HT nanodots to form Gal-dot and Man-dot probes. These multivalent Man-dots were capable of detecting particular overexpression of carbohydrate receptors on breast cancer cells, as well as produce ROS upon irradiation. The PDT results showed that although the treatment of breast cancer with irradiation or Man-dot alone could cause cell death, the integration of both Man-dot in the presence of a broadband light source (40 mW cm^-2^) lead to high quantity of dead cells in a concentration-dependent manner [77].

In another study anti HER-2 antibody, indocyanine green (ICG) and doxorubicin (DOX) were loaded onto polyethyleneimine (PEI)-coated perfluorocarbon double nanoemulsions (HIDPPDNEs) for the targeted photochemotherapy of HER-2 positive MDA-MB-453 breast cancer cells [78]. The proton sponge effect of PEI enabled the nanodroplets to escape endosome/lysosome intracellular uptake and so consequently their intercellular uptake and therapeutic effects were elevated. The adhesion efficiency of HIDPPDNEs for the breast cancer cells was 3-fold higher than the nanoemulsions without anti-HER-2 antibody. In addition, the production of ROS notably increase when 808 nm of near infrared radiation (NIR) light was applied and higher *in vitro* cell eradication was observed when twice the amount of nanodroplets encapsulated with ICG or DOX alone was applied [78].

In 2018, a thermosensitive drug delivery system composed of FA-PEG–DSPE and cRGD-PEG-PCL was studied to enhance the delivery of cisplatin and two PSs, namely ICG and Ce6 with the aid of two dual targeting agents (folic acid and peptide cRGD) [79]. The proposed nanoplatform was employed as a synergistic way for PDT, photothermal therapy (PTT) and chemotherapy of MCF-7 breast cancer cells. Gastric cancer SGC-7901 cell lines were also utilized to compare the results with MCF-7. The cellular uptake of dual targeting NPs for MCF-7 was more efficient than SGC-7901. Moreover, the cell group without NPs and 808 nm laser irradiation produced 9.1% apoptotic cell death, whilst 59.8% and 85.9% apoptotic cell death rates were reported for MCF-7 cells treated with only NPs and NPs in the presence of laser irradiation respectively [79].

Furthermore, an effective trimodal PDT/PTT/Chemotherapy system was accomplished by folate-conjugated polymer NPs [80]. Biodegradable poly (lactic-co-glycolic acid) (PLGA) was linked to ICG as a PS and photothermal agent, and carboplatin (CBP) [cis-diammine-(1,1-cyclobutanedicarboxylato)-platinum(II)], which interfered with DNA damage. Folic acid was also conjugated as a targeting ligand for folate receptor on breast cancer cells. The novel integrated delivery system was introduced to enhance the accumulation of drugs in tumour sites and to boost the antitumor efficiency of chem/PDT/PTT. It was suggested that the intercellular uptake of the proposed nanosystem was enhanced in MCF-7 breast cancer cells as a result of attaching the targeted NPs folate receptors. In fact, results showed that the NPs could localize in the cellular cytoplasm followed by the release of CBP/ICG in acidic pH tumor environment. The cell viability of unloaded PLGA-PEG NPs and blank PLGA-PEG-FOL NPs were reported to be 99.9% and 101%, which proved NP biocompatibility with no cytotoxic effect, whereas CBP/ICG formulated polymeric NPs demonstrated 2.5-fold higher cytotoxicity under 633 nm NIR light. With respect to *in vivo* experiments, the NPs obliterated tumour growth with NIR irradiation without remarkable damage to surrounding organs. Histopathological assessment of organ toxicity on kidney, liver and spleen demonstrated no toxicity due to NPs treatment which confirmed the safety and effectiveness of the nanosyystem for breast cancer trimodal PDT/PTT/chemotherapy [80].

Another chemo/photothermal/photodynamic combination therapy was developed by Jiao *et al.* with a nanoplatform for MDA-MB-231 breast cancer cells [81]. A novel coumarin-containing all-trans retinoic acid (AC) as an anti-tumour drug, a PS indocyanine green (ICG) dye-loaded polymer NPs with the targeted ligand cyclic (Arg-Gly-Asp-D-Phe-Lys) (cRGD) peptide were employed to prepare the nanoplatform of AC/ICG-TNPs. The nanosystem illustrated higher internalization in MDA-MB-231 cells compared to non-targeted AC/ICG-NPs. In addition, in connection with the low expression of αvβ3 in MCF-7 breast cancer cells, weaker cellular uptake was found for AC/ICG-TNPs. The treatment of MDA-MB-231 breast cancer cells with AC/ICG-TNPs mediated by ICG and 808 nm NIR irradiation produced significant ROS in contrast to free AC with laser or AC/ICG-TNPs group alone. More importantly, the low *in vitro* cell viability of 3.16% for treated cells with AC/ICG-TNPs under laser irradiation confirmed the high efficiency of the as-formed nanoplatform to eradicate the breast cancer cells, in particular MDAMB-231 breast cancer cells via the combinational therapies [81].

Polymeric NPs are sometimes coated with hydrophilic polyethylene glycol (PEG) and polysaccharides to preclude immune clearance by the reticuloendothelial system [82] and unwanted serum protein binding [83]. In a recent study by Wang *et al.,* multifunctional polysaccharide-based nanocomplexes (DOX/ALA-AHCS/HA^HER2^) were fabricated and consisted of cationic hydroxyethyl chitosan (HECS), anionic aldehyde-functionalized hyaluronic acid (AHA), and targeting ligand anti-HER-2 antibody-decorated AHA (HA^HER-2^) [84]. DOX and 5-ALA PS were then linked to the AHA as the core and then the cores were encapsulated with HECS shell and HA^HER2^ to fabricate DOX/ALA-AHCS/HA^HER2^ nanocomplexes. They proposed that the integration of ALA-PDT and DOX-mediated chemotherapy could eradicate the breast cancer MCF-7 cells. *In vitro* cellular uptake studies confirmed that the attachment of antibody onto the surface of the nanocomplexes strengthened the cellular uptake efficiency. DOX-AHCS/HA^HER-2^ produced similar cell viability to DOX/ALA-AHCS/HA^HER-2^ in the absence of 635 nm light, which was 48% and 47% respectively, while DOX/ALA-AHCS/HA^HER-2^ nanocomplexes demonstrated only 38% cell viability in the presence of light irradiation [84].

Overall, research in polymer therapeutics has grown tremendously in recent years enabling the controlled release of drugs over long periods of time, thanks to the breakthrough design of tailored nano polymers. In spite of continuous innovations, sustained efforts should be devoted towards the interference of polymer-based nanocarriers within biological systems and most importantly more *in vitro* studies are needed to scrutinize the effectiveness and safety of PS polymer nanosystems drug delivery abilities.

### Block copolymer micelles

In a study performed by Li *et al.*, Lyp-1 which is a breast cancer tumour homing peptide was utilized for the PDT/PTT and chemotherapy treatment of 4T1 breast cancer cells [85]. The dual component based micellar nanosystem was fabricated with the incorporation of two block polymers, methoxy poly(ethylene glycol)-poly(ε-caprolactone) (mPEG-PCL), and PCL-g-PEI-g-PEG with docetaxel (DTX), and co-loaded with NIR dye-IR820 as the PS. The tumour targeting delivery of DTX and IR820 was improved with the integration of the Lyp-1 peptides. The results showed that combination of the active targeting therapy mediated by Lyp-1 and PTT/PDT obliterated the breast cancer tumour cells after receiving 808 nm radiation. Additionally, this nanosystem was more efficient at inhibiting of the growth of breast cancer cells due to the presence of DTX/IR820-m-Lyp-1 micelles in comparison to DTX/IR820-m micelles treatment alone. *In vivo* investigations in 4T1 cancer model on Balb/C nude mice with Lyp-1 conjugated DTX micelles illustrated that chemotherapy/PDT/PTT co-therapy induced notable apoptotic breast cancer tumour cells death, inhibiting any further tumour growth and metastasis [85].

Triple-negative breast cancer (TNBC) is a subclass of breast cancer, which cannot be characterized or ligand targeted by overexpressed HER-2 or hormone receptors, such as oestrogen or progesterone [86]. Thus, hormonal or HER-2 receptor targeting therapeutics may not be utilized encourage effective nanosystems drug uptake [86]. Hence, studies by B. Feng *et al.*, attempted to combine chemotherapy and PDT in an attempt to construct a multifunctional prodrug NP for acid activatable fluorescence imaging to treat TNBC [87]. The prodrug NP comprised of an acid activatable PS (chlorin e6 (AC) derivative), the anticancer drug known as oxaliplatin (oxa) prodrug (hexadecyl-oxaliplatin trimethyleneamine, HOT) and a iRGD-conjugated phospholipid 1,2-distearoyl-sn-glycero-3-phosphoethanolamine-N-methoxy poly (ethylene glycol) (DSPE-PEG-iRGD) TNBC targeting agent. Cytotoxicity studies of the AC-loaded DSPE-PEG NPs noted that AC induced low cytotoxicity in the TNBC 4T1 cells without laser irradiation. However, the IC50 of the prodrug NP was 8.4 and 1.2-fold lower than that of Oxa and HOT, respectively. Furthermore, the study reported that the combination of AC-induced PDT and HOT-mediated therapy was more effective as it noted only 19% breast cancer cell viability, in comparison to PDT alone at 655 nm laser irradiation, which reported 53% viable cells and chemotherapy with NP@AC with about 58% cellular viability [87].

The application of copolymers was extended using two FDA-approved polymers in studies performed by Lee *et al.*, to fabricate anti-HER-2 indocyanine green (ICG) - doxorubicin (DOX) - encapsulated polyethylene glycol (PEG)-poly(lactic-co-glycolic acid) (PLGA) diblock (PEG-b-PLGA) copolymeric NPs (HIDPPNPs) for the phototherapy and chemotherapy of HER-2-overexpressing breast cancer cells [88]. Two breast cancer cell lines, namely MDA-MB-453/HER2(+) and MCF7/HER2(−) were analysed. Results reported that HIDPPNPs provided a 2-fold higher efficiency for MDA-MB-453/HER2(+) cells in comparison to MCF7/HER2(−) cells. Furthermore, HIDPPNPs illustrated improved thermal stability at concentrations ≥ 1 μM ICG equivalent and enhanced ROS generation under 808 nm laser irradiation, when compared to freely dissolved ICG. Moreover, the viability of the cells in the presence of the NPs and laser irradiation was lower than those without NIR exposure. In fact, the high cytotoxicity of the NPs with concentrations of 1 μM or higher ICG equivalent, was reported to be due to the fact that they incorporated high doses of DOX with the integration of photodynamic and photothermal effects, whereas the NPs with concentrations ≥ 0.5 μM ICG equivalent, only demonstrated moderate PDT effects with mild cytotoxicity [88].

Within another study the PDT treatment of TNBC using MDA-MB-231 cells was performed with a transferrin-targeted polymeric NPs [89]. PLGA–PEG-methoxy and PLGA–PEG-maleimide (blend polymer) NPs were used combined with a benzoporphyrin derivative monoacid (BPD) PS and hTf peptides to actively target TNBC transferrin receptors and called ANP. The BPD fluorescence signalling between the MDA-MB-231 cells and normal breast epithelial MCF 12A cells showed a significantly higher fluorescence, confirming that the active targeting of the NPs to target TNBC was successful. Within PDT studies on MDA-MB-231 TNBC cells using a 690 nm laser more cytotoxicity was noted in ANP treatments when compared to free BPD PS treatments at a concentration of 100 nM. Furthermore, PDT experiments using higher concentrations of ANP, noted a significant 95% cell death for targeted and passively targeted NPs (PLGA–PEG-BPD or PNP) which confirmed that the encapsulation of BPD into ANP or PNP had great advantages over BPD treatments alone [89].

Overall, in spite of the extensive application of micelles in drug delivery PDT applications for insoluble PSs, the potential dissociation of micelles and dilution below critical micelle concentration needs to be taken into consideration in order to prevent their unwanted side effects in physiological conditions [90, 91]. Nevertheless, regardless of such drawbacks, some nanomicelles such as paclitaxel, doxorubicin and cisplatin have successfully been introduced in clinical testing trials.

### Lipid-based NPs (liposomes)

Liposome-based NPs have been utilized over the years to enhance the overall concertation PSs in PDT treatments to be delivered to cancer cells with different physiochemical properties, since they have a very high loading capacity and so are considered valuable nanocarriers. Even though liposome-based NPs exhibit a short plasma half-life for within tumour drug uptake studies, it has been reported that active tumour-targeted liposomes are heightened tumoritropic associated carriers of PSs [92]. Furthermore, liposome NPs are able to encapsulate hydrophilic drugs within their aqueous core, while their lipid bilayers can carrying hydrophobic agents [3].

Studies by Yan *et al*. developed a multifunctional liposomal NP consisting of iRGD modified ICG-loaded liposomes (iRGD–ICG-LPs) for the PDT/PTT treatment of HUVECs, 4T1 and MCF-7 breast cancer cells [93]. The proposed nanoplatform demonstrated high tumor inhibition efficacy and tumor accumulation within PTT/PDT treatments. The results demonstrated that the iRGD–ICG-LPs displayed a far higher cellular uptake efficiency, when compared to ICG-LPs, in HUVECs and 4T1 cells, due to their overexpression of integrin αvβ3, when compared to MCF-7 cells, which express much lower levels of integrin αvβ3 for iRGD peptides to actively target. It is noteworthy to mention that the iRGD–ICG-LPs reported excellent biocompatibility with no cytotoxicity effects. The breast cancer tumor cells treated with iRGD–ICG-LPs and PDT reported elevated levels of singlet oxygen (^1^O_2_), with a respective 1.91-fold, 1.75-fold and 2.1-fold higher, than that of the breast cancer tumour cells treated with iRGD–ICG-LPs or laser light alone [93].

With reference to the above studies, it must be stated that so far very few studies have been conducted within the active targeting strategies using liposomal nanocarriers for the TPDT PS enhanced drug uptake in breast cancer cells and so this area of research requires further investigation. Studies by Allen and Cullis have noted that actively targeted liposomes are not necessarily more effective than non-targeted liposomes, although targeted liposomes have reported some merits in micrometastases, blood cancer and effective targeting abilities of a tumour’s vasculature nature [94].

### Biological nanoparticles (virus-like and albumin NPs)

Virus-like particles are a subclass of biological NPs for PDT which have a high stability, are small in size and have a large available manifold for easy modification [95, 96]. Studies by Cohen *et al.* produced MS2 bacteriophage capsids loaded with a meso-tetra-(4-N, N, N,-trimethylanilinium)-porphine (TMAP) PS in order to evaluate the targeted *in vitro* PDT effects in MCF-7 breast cancer cells and MCF-10 normal breast cells [97]. These MS2 bacteriophage capsids were also combined with a DNA aptamer that has repeating GGT motifs (G-quadruplex targeting aptamer, GTA), to target the nucleolin on the surface of MCF-7 cancer cells. The MS2–TMAP–GTA demonstrated no dark toxicity in the MCF-7 breast cancer cells nor within the normal breast MCF-10A control cells. Within *in vitro* PDT aptamer–virus–porphyrin (MS2–TMAP–GTA) based experiments, upon exposure to 630 nm laser light, significant photoinduced cytotoxicity in MCF-7 breast cells was noted. Additionally, the MS2 bacteriophage which was not modified with the targeting aptamer, provided no cytotoxicity in the presence or absence of the PS or laser irradiation. Furthermore, after PDT treatment 80% cell death was reported within MCF-7 breast cancer cells which were treated with the active targeting MS2–TMAP–GTA [97].

In order to overcome the low water solubility and poor ability of chlorine e6 to target tumours alone, studies by Phuong *et al.* investigated the utilization of albumin NPs with a nab™ (NP albumin-bound) technology. The nanoplatform consisted of bovine serum albumin (BSA), with beta-carotene as a carrier and Ce6 a photosensitizing agent (BSA-Ce6-BC-NPs) [98]. The albumin NPs reported a similar size to Abraxane* (~120 nm), with good physicochemical stability in the PDT treatments of 4T1 cells, which are a TNBC cell line. In the presence of free Ce6 and Ce6-BSA-BC-NPs followed by 660 nm laser irradiation, the cell viability was noted to be significantly lower in the breast cancer cells treated with Ce6-BSA-BC-NPs. Within the PDT *in vivo* treatment of 4T1 xenografted BALB/c nude mice, the Ce6-BSA-BC-NPs were found to be more significantly localized within the mouse tumours in comparison to their surroundings. More importantly, a significant 5 to 7-fold suppression in the tumour growth was observed in the breast cancer mouse models which were treated with Ce6-BSA-BC-NPs and laser irradiation in contrast to the free Ce6 controls [98].

Overall, the application of biological NPs should broadened the near future of breast cancer TPDT treatment, due to their imputable low toxicity, high biodegradability and biocompatibility, as well as their intrinsic ability to evade the immune system uncoated, while most other NPs required PEG coating to achieve a similar result [59].

## LIMITATIONS OF NANOCARRIERS

Although nanotechnology-based formulations have revolutionized cancer therapy, only a few of them have been introduced to the clinical testing. Physiochemical properties of nanoparticles such as their size, surface and biodistribution may affect their stability in physiological fluids and accumulation in nontarget organs [40, 99]. Furthermore, extra- and intracellular physiological barriers, hypersensitivity reaction induced by nanocarriers and osmotic pressure in cancer cells can lessen the amount of accumulated nanoparticles in the cancerous cells [40, 100].

It is anticipated that immobilization of anti-cancer drugs on the nanoparticles can enhance their biocompatibility, some studies have however shown their toxic effects on healthy cells and unfavourable interactions with biological entities [101, 102]. Moreover, since metastasized cells are too small, they produce lower microenvironment and EPR effect compared to solid tumours, the treatment of metastatic cancers is still challenging [100]. In addition to all the above, large scale-production of nanoformulations is a significant setback as physicochemical properties of them may vary for different batches [103].

Therefore, nanosaftey and physicochemical properties of nanoparticles along with drug and carrier pharmacokinetics have to be engineered before their clinical applications to improve biocompatibility and biodegradability and to avoid unwanted toxicity to health cells and release the nanocarriers at the intended cancer lesions.

## CONCLUSIONS AND FUTURE PERSPECTIVES

The prevalence of breast cancer urges researchers to investigate potent alternative strategies for its theranostic and combinative TPDT treatments. The integration of PSs with multifunctional organic NPs, not only can circumvent the associated drawbacks of conventional breast cancer treatments, but can also perfect the ability to utilize insoluble, toxic or unstable PSs. In addition, the surface modification of organic nanoplatforms with active targeting ligand agents (e. g. antibodies, folic acid, aptamer and small molecules) can enhance the overall accumulation of PS drugs into breast cancer tumour cells more selectively and so generate in turn higher levels of ROS, which overall improves the overall PDT treatment outcome effect.

In summation, this review highlighted the following aspects in relation to TPDT organic NPs breast cancer treatment: Polymeric/micelles NPs have been extensively researched, however more *in vivo* and clinical trials are needed to scrutinize effectiveness and safety in biological systems. With referral to block copolymer micelles, this review noted that these organic NPs have been extensively applied in TPDT applications and nanomicelles such as paclitaxel, doxorubicin and cisplatin have successfully been introduced in clinical testing trials, however drawbacks such as potential dissociation and dilution need to be considered. This review found that very few TPDT studies using liposomal NPs have been performed and so require further investigation, however they do tend to lack inherent effective active targeting abilities in relation to breast cancer. The findings of this review in relation to biological NPs (Virus-like and albumin NPs), it can most definitively be stated that they are the future of breast cancer TPDT treatment, due to their low toxicity, high biodegradability and biocompatibility, as well as their ability to evade the immune system components uncoated and so should be the core focus of up and coming TPDT breast cancer research.

In relation to this extensive review it is important for researchers to note the following considerations in relation to the TPDT of breast cancer using organic NPs; (1) it is of great importance to engineer biocompatible and biodegradable organic NPs towards blood cells and blood coagulation (2) dose-dependent and long-term toxicity of organic NPs should be appreciated in clinical trials, (3) absorption, biodistribution and excretion of organic NPs should be also taken into consideration via rigorous clinical tests.

In conclusion, it is fortunate that the arrival of organic nanocarriers and TPDT can be applied to usher breast cancer treatment in a new direction. Currently there are some organic NP-based therapeutics in clinical use for breast cancer treatment and with the many new avenues and opportunities offered by the unique properties of organic nanoplatforms, numerous other organic NPs are on the verge of entering to preclinical trials. Thus, researchers need to start paying more attention to organic nanoplatforms for the further development of actively TPDT for the conclusive treatment of breast cancer.

## SUPPLEMENTARY MATERIALS





## Abbreviations

Ad: adamantine
AHA: hyaluronic acid
AMPs: antimicrobial peptides
BCS: breast-conserving surgery
BODIPY: boron dipyrromethene
BPD: Benzoporphyrin derivative monoacid
BSA: bovine serum albumin
CBP: carboplatin
Ce6: Chlorin e6
DOX: doxorubicin
DTX: docetaxel
EGFR: epidermal growth factor receptors
FR: folate receptors
HECS: hydroxyethyl chitosan
HER: human epidermal receptor
HPPH: (2-[1-hexyloxyethyl]-2-devinyl pyropheophorbide-alpha
ICG: indocyanine green
LDL: low-density lipoprotein
mAb: Monoclonal antibodies
MB-SE: Methylene blue succinimidyl ester
MC540: merocyanin 540
NIR: near infrared
NPs: nanoparticles
PAA: polyacrylamide
PEG: polyethylene glycol
PEI: polyethyleneimine
PMRT: post mastectomy radiotherapy
PheoA: Pheophorbide A
PpIX: protoporphyrin IX
PS: photosensitizer
PSMA: Poly(styrene-co-maleic anhydride)
PTT: photothermal therapy
RB: rose bengal
ROS: reactive oxygen species
TfR: transferrin-receptors
tHPP: 5,10,15,20-tetrakis(4-hydroxyphenyl)-21H,23H-porphyrin
TMAP: meso-tetra-(4-N,N,N,-trimethylanilinium)-porphine
TNBC: Triple-negative breast cancer
TMAP: meso-tetra-(4-N,N,N,-trimethylanilinium)-porphine
TPDT: targeted photodynamic therapy
TPGS-PLA: D-α-tocopheryl polyethylene glycol 1000 succinate-poly(lactic acid)
ZnPc: zinc phthalocyanine
