# Supplementary material for: Review: Organic nanoparticle based active targeting for photodynamic therapy treatment of breast cancer cells
Source: Oncotarget. 2020 Jun 2;11(22):2120–36. doi: 10.18632/oncotarget.27596 (PMC7275783; doi:10.18632/oncotarget.27596)
Supplement: Supplementary file 1 [file oncotarget-11-2120-s001.pdf]

Review: Organic nanoparticle based active targeting for photodynamic therapy treatment of breast cancer cells

SUPPLEMENTARY MATERIALS

Highlights

- Breast cancer is the most frequent carcinoma among women.
- Photodynamic therapy (PDT) comprises of laser irradiaiton, a photosensitizer and oxygen to obliterate cancer cells.
- PDT is an alternative modality for breast cancer treatment with reduced side effects.
- Functionalized organic nanoparticles has a potential role in PDT treatment of breast cancer.
- Multifunctionalized nanomaterials with active targeting moieties ameliorate tumour drug uptake.

Supplementary Table 1: Chemical structures and absorption wavelengths of various photosensitizers utilized for active targeted PDT treatment of breast cancer. See Supplementary Table 1

REFERENCES

1. Zhang Q, Cai Y, Wang XJ, Xu JL, Ye Z, Wang S, Seeberger PH, Yin J. Targeted photodynamic killing of breast cancer cells employing heptamannosylated β-cyclodextrin-mediated nanoparticle formation of an adamantane-functionalized BODIPY photosensitizer. ACS Appl Mater Interfaces. 2016; 8:33405–33411. https://doi.org/10.1021/acsami.6b13612. [PubMed]
2. Zhang Q, Cai Y, Li QY, Hao LN, Ma Z, Wang XJ, Yin J. Targeted Delivery of a Mannose-Conjugated BODIPY Photosensitizer by Nanomicelles for Photodynamic Breast Cancer Therapy. Chem: Eur J. 2017; 23:14307–14315. https://doi.org/10.1002/chem.201702935. [PubMed]
3. Bharathiraja S, Moorthy MS, Manivasagan P, Seo H, Lee KD, Oh J. Chlorin e6 conjugated silica nanoparticles for targeted and effective photodynamic therapy. Photodiagn Photodyn Ther. 2017; 19:212–220. https://doi.org/10.1016/j.pdpdt.2017.06.001. [PubMed]
4. Yu X, Gao D, Gao L, Lai J, Zhang C, Zhao Y, Zhong L, Jia B, Wang F, Chen X. Inhibiting metastasis and preventing tumor relapse by triggering host immunity with tumor-targeted photodynamic therapy using photosensitizer-loaded functional nanographenes. ACS Nano. 2017; 11:10147–10158. https://doi.org/10.1021/acsnano.7b04736. [PubMed]
5. Gao F, Bai L, Feng X, Tham HP, Zhang R, Zhang Y, Liu S, Zhao L, Zheng Y, Zhao Y. Remarkable *In Vivo* Nonlinear Photoacoustic Imaging Based on Near-Infrared Organic Dyes. Small. 2016; 12:5239–5244. https://doi.org/10.1002/smll.201602121. [PubMed]
6. Yu Z, Xia Y, Xing J, Li Z, Zhen J, Jin Y, Tian Y, Liu C, Jiang Z, Li J. Y 1-receptor–ligand-functionalized ultrasmall upconversion nanoparticles for tumor-targeted trimodality imaging and photodynamic therapy with low toxicity. Nanoscale. 2018; 10:17038–17052. https://doi.org/10.1039/C8NR02387E. [PubMed]
7. Lee YH, Chang DS. Fabrication, characterization, and biological evaluation of anti-HER2 indocyanine green-

doxorubicin-encapsulated PEG-b-PLGA copolymeric nanoparticles for targeted photochemotherapy of breast cancer cells. Sci Rep. 2017; 7:46688. https://doi.org/10.1038/srep46688. [PubMed]

8. Hah HJ, Kim G, Lee YEK, Orringer DA, Sagher O, Philbert MA, Kopelman R. Methylene blue-conjugated hydrogel nanoparticles and tumor-cell targeted photodynamic therapy. Macromol Biosci. 2011; 11:90–99. https://doi.org/10.1002/mabi.201000231. [PubMed]
9. Eichwurzel I, Stiel H, Röder B. Photophysical studies of the pheophorbide a dimer. J Photochem Photobiol B, Biol. 2000; 54:194–200. https://doi.org/10.1016/S1011-1344(00)00016-6. [PubMed]
10. Weijer R, Broekgaarden M, Kos M, van Vught R, Rauws EA, Breukink E, van Gulik TM, Storm G, Heger M. Enhancing photodynamic therapy of refractory solid cancers: Combining second-generation photosensitizers with multi-targeted liposomal delivery. J Photochem Photobiol C Photochem Rev. 2015; 23:103–131. https://doi.org/10.1016/j.jphotochemrev.2015.05.002.
11. Islam MA, Haither MJ, Khan I, Islam M. Optical and structural characterization of TiO2 nanoparticles. IOSR J Electric Electron Eng. 2012; 3:18–24. https://pdfs.semanticscholar.org/6e42/01671158f2ef85deb3e7c8e62523607e16d9.pdf.
12. Xu W, Qian J, Hou G, Wang Y, Wang J, Sun T, Ji L, Suo A, Yao Y. A dual-targeted hyaluronic acid-gold nanorod platform with triple-stimuli responsiveness for photodynamic/photothermal therapy of breast cancer. Acta Biomater. 2019; 83:400–413. https://doi.org/10.1016/j.actbio.2018.11.026. [PubMed]
13. Jadia R, Kydd J, Rai P. Remotely Phototriggered, Transferrin-Targeted Polymeric Nanoparticles for the Treatment of Breast Cancer. Photochem Photobiol. 2018; 94:765–774. https://doi.org/10.1111/php.12903. [PubMed]
14. Narsireddy A, Vijayashree K, Irudayaraj J, Manorama SV, Rao NM. Targeted *in vivo* photodynamic therapy with epidermal growth factor receptor-specific peptide linked nanoparticles. Int J Pharm. 2014; 471:421–429. https://doi.org/10.1016/j.ijpharm.2014.05.063. [PubMed]
15. Zhang Y, Zhang H, Wang Z, Jin Y. pH-Sensitive graphene oxide conjugate purpurin-18 methyl ester photosensitizer nanocomplex in photodynamic therapy. New J Chem. 2018; 42:13272–13284. https://doi.org/10.1039/C8NJ00439K.
16. Ng KK, Takada M, Jin CC, Zheng G. Self-sensing porphysomes for fluorescence-guided photothermal therapy. Bioconjugate Chem. 2015; 26:345–351. https://doi.org/10.1021/bc500595d. [PubMed]
17. Cohen BA, Bergkvist M. Targeted *in vitro* photodynamic therapy via aptamer-labeled, porphyrin-loaded virus capsids. J. Photochem. Photobiol. B, Biol. 2013; 121:67–74. https://doi.org/10.1016/j.jphotobiol.2013.02.013. [PubMed]
18. Cohen BA, Kaloyeros AE, Bergkvist M. MS2 bacteriophage as a delivery vessel of porphyrins for photodynamic therapy. Optical Methods for tumort treatment and detection: mechanisms and techniques in photodynmmic therapy XX, International Society for Optics and Photonics. 2011; 2011:788614. https://doi.org/10.1117/12.875202.
19. Feng Y, Wu Y, Zuo J, Tu L, Que I, Chang Y, Cruz LJ, Chan A, Zhang H. Assembly of upconversion nanophotosensitizer *in vivo* to achieve scatheless real-time imaging and selective photodynamic therapy. Biomaterials. 2019; 201:33–41. https://doi.org/10.1016/j.biomaterials.2019.02.015. [PubMed]
