# Supplementary material for: Review: Organic nanoparticle based active targeting for photodynamic therapy treatment of breast cancer cells
Source: Oncotarget. 2020 Jun 2;11(22):2120–36. doi: 10.18632/oncotarget.27596 (PMC7275783; doi:10.18632/oncotarget.27596)
Supplement: Supplementary file 2 [file oncotarget-11-2120-s002.pdf]

Supplementary Table 1: Chemical structures and absorption wavelengths of various photosensitizers utilized for active targeted PDT treatment of breast cancer

Type of PS	PS	Absorption wavelength (nm)	Chemical structure	Ref.
BODIPY-type	Boron dipyrromethene (BODIPY)	665	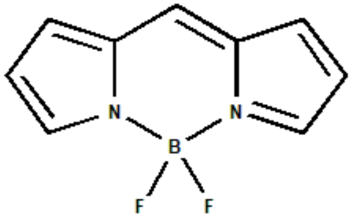	[1, 2]
Chlorines	Chlorin e6 (Ce6)	670	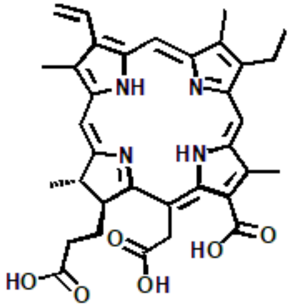	[3]
	(2-[1-hexyloxyethyl]-2-devinyl pyropheophorbide-alpha (HPPH)	665	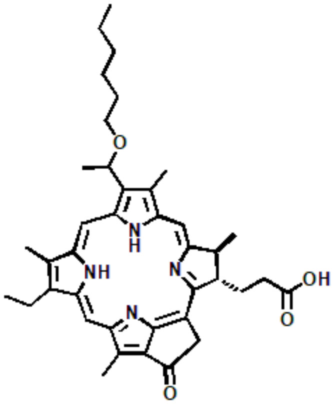	[4]

	IR820 dye	819	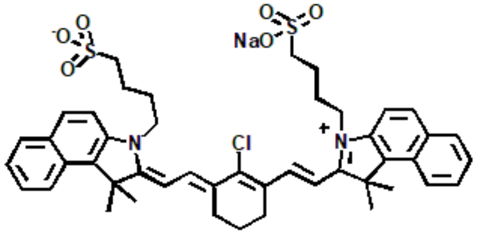	[5]
Cyanines	Merocyanin 540 (MC 540)	540	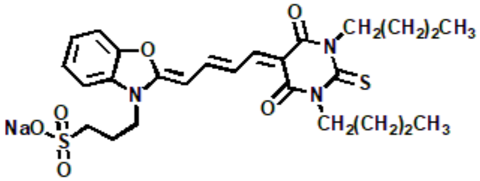	[6]
	Indocyanine green (ICG)	780	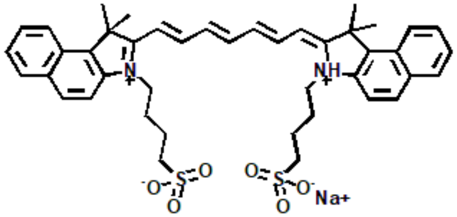	[7]
Dye-type	Methylene blue succinimidyl ester (MB-SE)	666	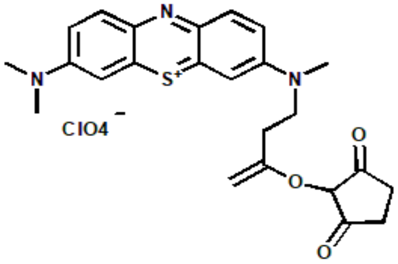	[8]

Herbal extracts	Pheophorbide A (PheoA)	667	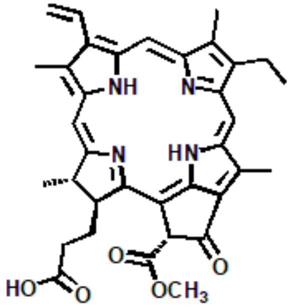	[9]
Metalophthalocyanines	Zinc phthalocyanine (ZnPc)	674	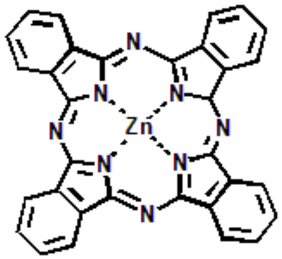	[10]
Nanostructured metal oxides	TiO_2 NPs	423	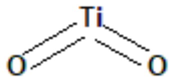	[11]
Porphyrins	Protoporphyrin IX (PpIX)	635	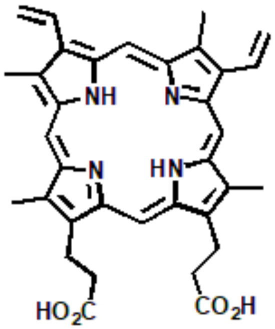	[12]

Benzoporphyrin derivative monoacid (BPD)	690	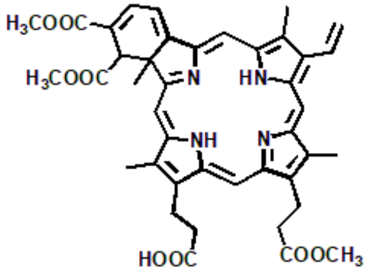	[13]
5,10,15,20-tetrakis(4-hydroxyphenyl)-21H,23H-porphyrin (tHPP)	420	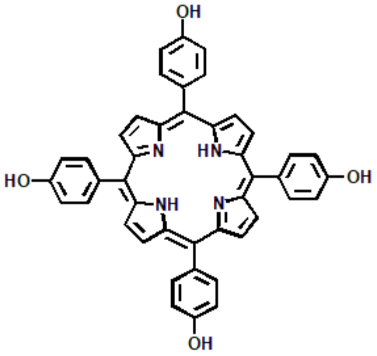	[14]
Purpurin-18	699	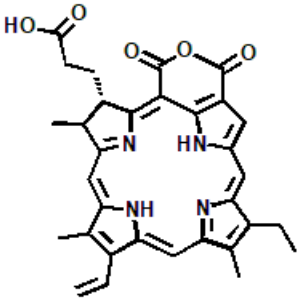	[15]

	Pyrolipid	665	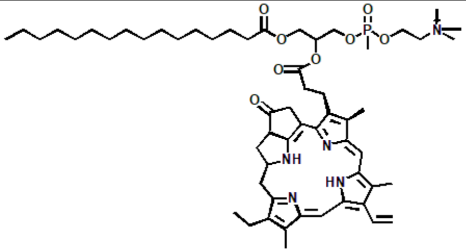	[16]
	Meso-tetra-(4-N,N,N,-trimethylanilinium)-porphine (TMAP)	630	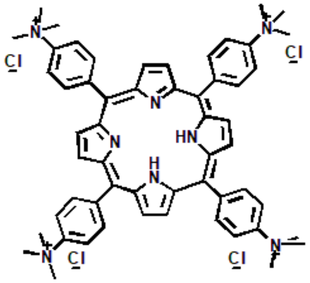	[17, 18]
Xanthenes	Rose bengal (RB)	562	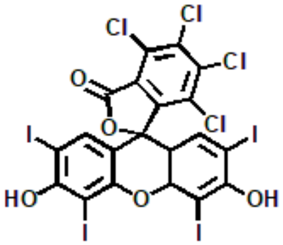	[19]
